# 

**Published:** 1912-08

**Authors:** 


### CONTENTS.

ORIGINAL COMMUNICATIONS:—

The Value of the Roentgen Rays in Diagnosis of Kidney Disease. By John S. Derr, Atlanta, Ga. ---------- 228

Visceroptosis. By J. Norment Baker, B. A., M. D., Montgomery, Ala. 243

Injuries to the Spinal Cord. By Frank K. Boland, M. D., Atlanta, Ga. 252

Gastric Neuroses. By William J. Mallory, M. D., Washington, D. C. - 257

EDITORIALS ---------- 268

NEWS AND NOTES ---------- 271

BOOK REVIEWS ---------- 285

